# Cross-cultural adaptation and validation of the Michigan Hand Outcomes Questionnaire (MHQ) for Brazil: validation study

**DOI:** 10.1590/1516-3180-2014-1326701

**Published:** 2014-09-02

**Authors:** Sandra Mara Meireles, Jamil Natour, Daniel Alberton Batista, Mayara Lopes, Thelma Larocca Skare

**Affiliations:** I PT, PhD. Physiotherapist, Rheumatology Division, Escola Paulista de Medicina, Universidade Federal de São Paulo (EPM-Unifesp), São Paulo, Brazil; II MD, PhD. Associate Professor, Rheumatology Division, Escola Paulista de Medicina, Universidade Federal de São Paulo (EPM-Unifesp), São Paulo, Brazil; III PT, BSc. Physiotherapist, Rheumatology Unit, Hospital Universitário Evangélico de Curitiba (HUEC), Curitiba, Brazil; IV MD, PhD. Head of Rheumatology Unit, Hospital Universitário Evangélico de Curitiba (HUEC), and Associate Professor, Discipline of Rheumatology, Faculdade Evangélica do Paraná, Curitiba, Brazil

**Keywords:** Hand, Questionnaires, Arthritis, rheumatoid, Validation studies [publication type], Wrist, Mãos, Questionários, Artrite reumatoide, Estudos de validação, Punho

## Abstract

**CONTEXT AND OBJECTIVE:**

Rheumatoid arthritis is a chronic systemic disease that causes joint damage. A variety of methods have been used to evaluate the general health status of these patients but few have specifically evaluated the hands. The objective of this study was to translate, perform cultural adaptation and assess the validity of the Michigan Hand Outcomes Questionnaire for Brazil.

**DESIGN AND SETTING:**

Validation study conducted at a university hospital in Curitiba, Brazil.

**METHODS:**

Firstly, the questionnaire was translated into Brazilian Portuguese and back-translated into English. The Portuguese version was tested on 30 patients with rheumatoid arthritis and proved to be understandable and culturally adapted. After that, 30 patients with rheumatoid arthritis were evaluated three times. On the first occasion, two evaluators applied the questionnaire to check inter-rater reproducibility. After 15 days, one of the evaluators reassessed the patients to verify intra rater reproducibility. To check the construct validity at the first assessment, one of the evaluators also applied other similar instruments.

**RESULTS:**

There were strong inter and intra rater correlations in all the domains of the Michigan Hand Outcomes Questionnaire. Cronbach's alpha was higher than 0.90 for all the domains of the questionnaire, thus indicating excellent internal validity. Almost all domains of the questionnaire presented moderate or strong correlation with other instruments, thereby showing good construct validity.

**CONCLUSION:**

The Brazilian Portuguese version of the Michigan Hand Outcomes Questionnaire was translated and culturally adapted successfully, and it showed excellent internal consistency, reproducibility and construct validity.

## Abstract (pt)

**CONTEXTO E OBJETIVO:**

Artrite reumatoide é uma doença crônica, sistêmica, que provoca danos articulares. Diversos métodos têm sido usados para avaliar o estado geral de saúde desses pacientes, mas poucos avaliam especificamente as mãos. O objetivo deste estudo foi traduzir, realizar a adaptação cultural e testar a validade do Michigan Hand Outcomes Questionnaire para o Brasil.

**TIPO DE ESTUDO E LOCAL:**

Estudo de validação feito em um hospital universitário em Curitiba, Brasil.

**MÉTODOS:**

Na primeira etapa, o questionário foi traduzido para o português do Brasil e traduzido de volta para o inglês. A versão em português foi testada em 30 pacientes com artrite reumatoide e mostrou-se compreensível e adaptada culturalmente. Na segunda etapa, 30 pacientes com artrite reumatoide foram avaliados três vezes. Na primeira vez, dois avaliadores aplicaram o questionário para verificação da reprodutibilidade interavaliadores. Após 15 dias, um dos avaliadores reavaliou os pacientes para verificação da reprodutibilidade intra-avaliadores. Para verificar a validade construtiva, na primeira avaliação, um dos avaliadores aplicou também outros instrumentos de avaliação similares.

**RESULTADOS:**

Foram observadas fortes correlações interavaliadores e intra-avaliadores em todos os domínios do Michigan Hand Outcomes Questionnaire. O alfa de Cronbach foi maior que 0.90 para todos os domínios, indicando ótima validade interna. A maioria dos domínios do questionário apresentou correlação moderada ou forte com outros instrumentos, determinando boa validade construtiva.

**CONCLUSÃO:**

A versão brasileira do Michigan Hand Outcomes Questionnaire foi traduzida, adaptada culturalmente com sucesso e apresentou ótima consistência interna, reprodutibilidade e validade construtiva.

## INTRODUCTION

Rheumatoid arthritis (RA) is a chronic autoimmune disease that affects all synovial joints, with progressive and irreversible joint destruction.^1^ Hand dysfunction and deformities are some of the most common manifestations of this disease and they are an important cause of morbidity, since they interfere with individuals' capacity to perform self-care, work productivity and social interactions.^2^^,^ 3 A variety of methods have been used to evaluate general health status in RA patients but few have been designed to access particularly the hands.^4^

The Michigan Hand Outcomes Questionnaire (MHQ) is a hand evaluation instrument that was conceived at the University of Michigan in 1998 using psychometric principles.^5^ This is a self-administered instrument that has 37 items that evaluate six domains: overall hand function, activities of daily living, work performance, pain, esthetics and patient satisfaction with hand function. This instrument is intended for use among individuals with hand and wrist conditions and injuries, including arthritis. The right and left hand can be evaluated separately. It takes nearly 15 minutes to complete and has been found to be valid and reliable for measuring hand function in RA patients.^4^^-^ 6 The MHQ has been also translated into other languages such as German,^7^ Turkish^8^ and Korean.^9^

## OBJECTIVE

The objectives were to translate and cross-culturally adapt the original MHQ to produce a Brazilian Portuguese version, and to assess its validity.

## METHODS

### Type of study and sample

This was a validation study that was approved by the Research Ethics Committee of the Evangelic Society of Curitiba, Paraná, and all participants gave their signed consent prior to the interview.

Data were gathered between September 2010 and September 2012 and 60 patients were included: 30 patients to test the understanding of the initial version (used for translation and cultural adaptation) and another 30 patients to test reproducibility and construct validity. This was a convenience sample. The number used was chosen in accordance with the guidance of Beaton et al.,^10^ which has been used in other published papers to test the cultural validation and reproducibility of other questionnaires.^11^^-^ 14

All the patients investigated were users of the public healthcare system (Brazilian National Health System, SUS).

### Translation and cultural adaptation

Two independent native speakers of Brazilian Portuguese who were fluent in English translated the original MHQ from English to Brazilian Portuguese in the manner recommended by Guillemin et al.^15^ and by the guidelines of the American Academy of Orthopedic Surgeons Outcome Committee.^10^ This translation was reviewed by a committee composed of two rheumatologists and a physiotherapist, which then reached a consensus regarding the Brazilian Portuguese version. This version was then backtranslated to English by two native English speakers who did not know the initial questionnaire. This version was compared with the original version and was demonstrated to be semantically equivalent.

This version of the MHQ in Brazilian Portuguese (which was considered to be the test version) was administered to 30 patients with rheumatoid arthritis, who were selected from the Rheumatology Outpatient Clinic of the Evangelical University Hospital in Curitiba, Paraná, taking into account the American College of Rheumatology (ACR) classification criteria.^16^ We included patients of both genders, between 18 and 60 years of age, who were chosen according to appointment order and their willingness to participate in the study. All the patients had RA with hand and wrist involvement and their disease had been diagnosed more than one year earlier. We excluded patients with other associated rheumatic diseases, other upper limb musculoskeletal conditions, previous hand or wrist surgery, previous hand or wrist trauma in the last month or neurological diseases.

With regard to cultural equivalence, the patients' degree of understanding was measured by a yes/no answer to the question: "Do you understand what is being asked for"? Any items that were not understood by 20% of the respondents would be revised by the expert committee and the new version would be retested on 30 patients. The proportion of 20% was defined in accordance with what had been used in previous, similar published papers.^12^^,^ 13 ^,^ 17 ^,^ 18

### Reproducibility

A new group of 30 patients was selected using the same inclusion and exclusion criteria, after the MHQ had been tested and semantic and cultural equivalence had been attained. These patients were evaluated three times. In the first interview, two examiners administered the questionnaire on the same day to check inter-rater reproducibility. In the second interview, which was conducted 15 days later, one of the first reviewers reapplied the MHQ with the intention of verifying the inter-assessment reproducibility. The internal consistency of the multi-item subscales was assessed.

### Construct validity

The construct validity was tested in the first interview through simultaneous application of the Disability of the Arm, Shoulder and Hand questionnaire (DASH),19 Visual Analogue Scale (VAS) of pain,^20^ COCHIN Hand Function Scale^13^ and Health Assessment Questionnaire (HAQ).^21^ These instruments had already been validated for Brazilian Portuguese and they assess dysfunctions of the upper limbs (DASH and COCHIN) and general function among rheumatoid arthritis patients (HAQ).

### Statistical analysis 

We used descriptive statistical analysis showing the mean and standard deviation of the data. Intraclass correlation coefficient (ICC) evaluation and Bland-Altman analysis were used to assess the interobserver and intra-observer reproducibility. Internal consistency was assessed by means of Cronbach's alpha test. The Spearman correlation test was used to investigate the construct validity.

Calculations were done with the aid of the GraphPad Prism 6.0 software (GraphPad Software, Inc, La Jolla, CA, USA) and SPSS 17.0 (Chicago, IL, USA).

## RESULTS

Thirty patients diagnosed with RA according to the ACR criteria^16^ were selected and participated in the initial phase of the interview. Over 80% of the patients understood all the questions in the questionnaire. The translation of the MHQ into Brazilian Portuguese, with cultural equivalence, is attached (Appendix 1). Another 30 patients were evaluated to verify the reproducibility, internal consistency and construct validity.

There were no losses in applying the protocol. All the patients who agreed to participate completed the whole evaluation, and the evaluator checked whether each questionnaire had been completed before releasing the patient. About 30% of the patients who were invited to participate in the study did not accept the invitation and thus were not included. Table 1 shows the demographic and clinical data on the participating patients.

**Table 1:** Clinical and demographic data on patients interviewed during the reproducibility phase (n = 30)

Variable	Frequency
Age (years)	49.9 ± 9.3^*^
Gender	
Female (%)	25 (83.4)
Male (%)	5 (16.6)
Ethnic background	
Caucasian (%)	22 (74)
Afro-descendent (%)	8 (26)
Disease duration (years)	11 ± 8.9^*^
Formal education (years)	7.1 ± 4.5^*^
Dominant hand	
Right (%)	25 (83.4)
Left (%)	5 (16.6)
Daily difficulties (%)	
Carrying weight	5 (16.6)
Manual activities	16 (53.3)
Domestic work	8 (26.6)
No difficulty	1 (0.3)

* Mean ± standard deviation

n = number.

Table 2 shows that there were strong correlations between the results obtained in the intra and inter-examiner evaluations, with ICC ranging from 0.841 to 0.967 in the intra-examiner evaluation and ICC ranging from 0.753 to 0.921 in the inter-examiner evaluation (95% confidence interval). No patient had medication prescriptions chaged in the interval between test and retest. Only in the field of ADLs (activities of daily living) relating to the right hand was the correlation found to be lower, i.e. 0.611, which is a moderate inter-rater association. Also in Table 2, it can be seen that Cronbach's alpha was greater than 0.908 for all areas, thus indicating that the questionnaire had good internal consistency.

**Table 2:** Inter and intra-examiner reproducibility and internal consistency of Michigan Hand Outcomes Questionnaire domains

Domain	A1 Mean ± SD	A2 Mean ± SD	R2 Mean ± SD	ICC A1XA2	ICC A2XR2	Cronbach’s alpha
RH function	52.2 ± 19.0	53.3 ± 21.5	55.3 ± 24.8	0.915	0.863	0.908
LH function	54.2 ± 24.0	54.7 ± 24.9	54.7 ± 23.0	0.908	0.875	0.941
RH ADL	73.3 ± 21.2	73.5 ± 20.8	72.5 ± 25.1	0.901	0.611	0.871
LH ADL	72.2 ± 26.3	71.0 ± 26.2	66.5 ± 28.7	0.841	0.783	0.939
BH ADL	62.5 ± 27.6	62.1 ± 27.5	63.9 ± 24.1	0.967	0.818	0.930
Work	46.0 ± 29.3	46.8 ± 25.8	45.0 ± 29.2	0.918	0.753	0.969
RH pain	49.2 ± 25.3	47.0 ± 27.1	48.7 ± 29.6	0.929	0.885	0.908
LH pain	47.8 ± 30.8	41.5 ± 32.7	47.7 ± 30.6	0.944	0.826	0.941
RH esthetics	44.0 ± 26.8	44.6 ± 29.0	43.5 ± 31.6	0.929	0.921	0.864
LH esthetics	48.8 ± 31.2	48.8 ± 33.1	45.4 ± 34.0	0.919	0.905	0.925
RH satisfaction	44.2 ± 29.3	44.2 ± 29.8	47.4 ± 30.0	0.883	0.786	0.940
LH satisfaction	47.8 ± 32.0	49.9 ± 34.4	44.3 ± 33.0	0.937	0.876	0.939

A1 = first evaluation

**A1XA2 :**= inter-rater evaluation

A2 = second evaluation

**A2XR2 :**= intra-rater evaluation

ADL = activities of daily living

BH = both hands

ICC = intraclass correlation

LH = left hand

R2 = re-evaluation

RH = right hand

SD = standard deviation

### Table 3 and Figure 1 show the strong intra and inter-rater correlation for both hands in the final outcome from the MHQ.

**Table 3:** Inter and intra-examiner reproducibility and internal consistency of general results from the MHQ (Michigan Hand Outcomes Questionnaire)

Domain	A1 mean ± SD	A2 mean ± SD	R2 mean ± SD	ICC A1XA2	ICC A2XR2	Cronbach’s alpha
Right-hand general results	51.9 ± 19.6	52.5 ± 20.0	51,7 ± 23.4	0.976	0.917	0.868
Left-hand general results	53.8 ± 23.7	55.2 ± 23.8	52.7 ± 24.1	0.980	0.936	0.914

A1 = first evaluation

**A1XA2 :**= inter rater evaluation

A2 = second evaluation

**A2XR2 :**= intra rater evaluation

ICC = intraclass correlation

R2 = re-evaluation

SD = standard deviation

**Figure 1:**
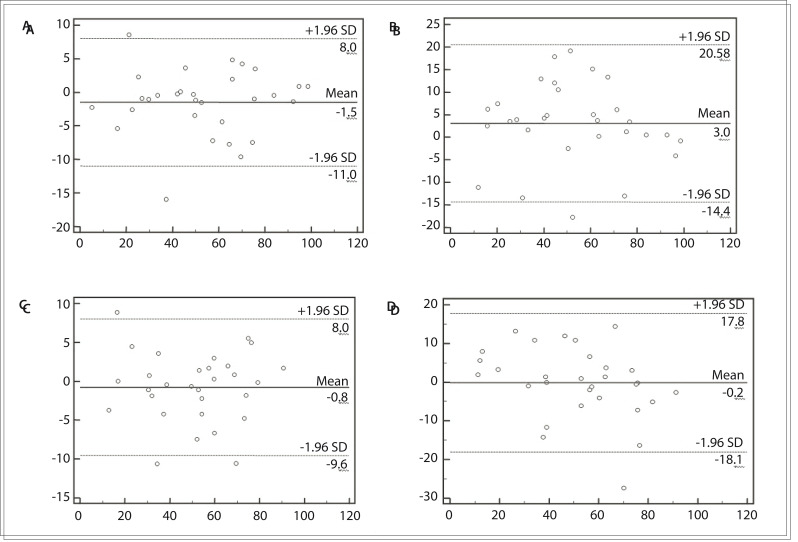
Bland-Altman graphs with reproducibility and standard deviations (SD). (A) Left hand: reproducibility between first and second evaluators (interclass); (B) Left hand: reproducibility between first evaluator and re-evaluation (intraclass); (C) Right hand: reproducibility between first and second evaluators (interclass); (D) Right hand: reproducibility between first evaluator and re-evaluation (intraclass).

Table 4 demonstrates the correlation between the domains of the MHQ and other instruments such as HAQ, DASH, DASH Work, COCHIN and VAS for pain. Taking into account the dominant hand, it can be seen that for all items of the MHQ, moderate and strong correlations (r_s_ ranging from -0.41 to -0.89) were found.

**Table 4:** Correlations* between MHQ domains obtained in the first evaluation (reproducibility phase) and the HAQ, DASH, DASH Work, COCHIN Hand Function Scale and Visual Analogue Scale for pain, to assess construct validation.

	HAQ		DASH		DASH Work		COCHIN		VAS	
	r_s_	P-value	r_s_	P-value	r_s_	P-value	r_s_	P-value	r_s_	P-value
Function	-0.62	0.0002	-0.67	< 0.0001	-0.73	< 0.0001	-0.56	0.0010	-0.60	0.0004
ADL	-0.64	0.0001	-0.70	< 0.0001	-0.59	0.0009	-0.81	< 0.0001	-0.43	0.0157
BH ADL	-0.74	< 0.0001	-0.84	< 0.0001	-0.73	< 0.0001	-0.89	< 0.0001	-0.55	0.0013
Work	-0.72	< 0.0001	-0.69	< 0.0001	-0.79	< 0.0001	-0.59	0.0005	-0.50	0.0043
Pain	0.62	0.0002	0.65	< 0.0001	0.62	0.0003	0.51	0.0033	0.75	< 0.0001
Esthetics	-0.44	0.0147	-0.41	0.0239	-0.43	0.0204	-0.50	0.0041	-0.52	0.0029
Satisfaction	-0.47	0.0077	-0.55	0.0015	-0.64	0.0002	-0.48	0.0071	-0.58	0.0006

ADL = activities of daily living

BH = both hands

DASH = Disability of the Arm, Shoulder and Hand Questionnaire

HAQ = Health Assessment Questionnaire

MHQ = Michigan Hand Outcomes Questionnaire

VAS = Visual Analogue Scale

* All correlations were performed using the Spearman test. Spearman rs < 0.3 was considered to be a weak correlation; 0.3 to 0.6, moderate; and > 0.6, strong.

## DISCUSSION

RA is a chronic systemic disease that causes joint damage especially in the wrist and small joints of the hands. Decreased joint mobility, reduced grip strength and deformities occur early in the disease and are some of the major determinants of the disease outcome.^22^ Hand dysfunction is an important cause of disability in RA cases, and therefore it is important to evaluate hand joint damage in order to institute effective treatment.^23^

A growing number of questionnaires for evaluating hand function and the impact of RA on patients' quality of life have been introduced.^24^^,^ 25 What a patient feels can be expressed in different ways, since discomfort, pain and disability are individual and subjective concepts.^26^^,^ 27 Therefore, these questionnaires allow measurement of symptoms more objectively and enable comparison of these data between different researchers or by a single researcher, at different times of the disease in the same patient.^28^^,^ 29 There are two possible ways to obtain a questionnaire that can be used in a certain language: creation of a questionnaire for a particular ethnic group; or translation and validation of a questionnaire that was previously developed for another language.^15^ This second option, in addition to being more economical in terms of time and resources, allows comparison of data obtained in different countries.

The MHQ measures individuals' perceptions of their hands in terms of function, appearance, pain and satisfaction. These last three items provide an advantage for this questionnaire over the COCHIN Rheumatoid Hand Disability scale, which does not include them. Pain control and esthetics have been demonstrated to be important motivators for surgical interventions in RA patients.^30^ The MHQ also discriminates between the right and left hand in each performance domain, a distinction that is not offered by the Disability of the Arm, Shoulder and Hand questionnaire (DASH).^19^^,^ 25 DASH is also a general arm instrument.^19^

We present here a Brazilian Portuguese version for MHQ. We have followed the validation process proposed in the guidelines of the American Academy of Orthopedic Surgeons Outcome Committee.^10^ The steps of translation and back-translation did not show any major linguistic or cultural discrepancies. Furthermore, the internal consistency of each item in all domains was high (Cronbach's alpha ranging from 0.86 to 0.96). 

In this study, we chose a test-retest interval of two weeks. RA is a chronic disease and we believed that over a two-week period, no important changes to the disease status would occur but that this would be long enough for a patient not to recall the content of the instrument from the first interview. None of the patients had any changes in medication over this interval. Both the intraclass correlation (ranging from 0.84 to 0.96) and the interclass correlation (ranging from 0.61 to 0.92) were high, as can be seen in Figure 1.

Concerning the construct validity, we compared the Brazilian Portuguese version of MHQ with DASH, COCHIN, VAS for pain and HAQ. We found moderate to high correlations between these instruments and most of the MHQ domains, except for the following: esthetics, which showed weak correlations with HAQ, DASH and DASH Work; ADL, which showed a weak correlation with VAS; and satisfaction, which showed a weak correlation with HAQ. Since the MHQ is the only instrument that evaluates esthetics and satisfaction, this explains the weak correlation found.

One weakness of this study is that only 30 patients were included in each phase. However, this disadvantage was minimized by achieving a Cronbach's alpha for internal consistency that was higher than 0.90. Calculating Cronbach's alpha in future studies using this tool will certainly help support its validity.

Another weakness to be taken into account is the lack of economic profile information for the patients in our dataset. Although this does not affect the validation of the questionnaire, it does preclude comparisons of this characteristic in future studies.

## CONCLUSION

We conclude that the Brazilian Portuguese version of the MHQ was successfully translated and adapted, with very good internal consistency, reliability and construct validity.
